# Pulsed synchrony regulation of intraocular and cerebrospinal fluid pressure: a novel paradigm for glaucoma pathogenesis and treatment

**DOI:** 10.3389/fmed.2026.1781347

**Published:** 2026-03-24

**Authors:** Bin Lin, Duan-Qing Huang, Ting-Ting Li, Wei Liang, Meng Xu, Dong-Kan Li

**Affiliations:** 1 Xiamen Eye Center and Eye Institute of Xiamen University, School of Medicine, Xiamen, China; 2 Xiamen Clinical Research Center for Eye Diseases, Xiamen, Fujian, China; 3 Xiamen Key Laboratory of Ophthalmology, Xiamen, Fujian, China; 4 Fujian Key Laboratory of Corneal & Ocular Surface Diseases, Xiamen, Fujian, China; 5 Xiamen Key Laboratory of Corneal & Ocular Surface Diseases, Xiamen, Fujian, China; 6 Translational Medicine Institute of Xiamen Eye Center of Xiamen University, Xiamen, Fujian, China

**Keywords:** cerebrospinal fluid pressure (CSFP), dynamic imbalance, glaucoma, intraocular pressure (IOP), trans-lamina cribrosa pressure difference (TLCPD)

## Abstract

Glaucoma, the leading cause of irreversible blindness worldwide, is characterized by the progressive loss of retinal ganglion cells (RGCs) and their axons. While elevated intraocular pressure (IOP) is a core risk factor, the pathogenesis of normal-tension glaucoma (NTG) remains unclear, as static IOP is within the normal range. Based on circadian fluctuations of IOP and cerebrospinal fluid pressure (CSFP), and the pressure-dependent function of the ocular glymphatic system, we propose the “dynamic trans-lamina cribrosa pressure difference (TLCPD) imbalance” hypothesis. This hypothesis posits that optic nerve damage may stem from abnormal pulse synchrony between IOP and CSFP (phase mismatch, amplitude mismatch, or abnormal frequency) rather than static TLCPD elevation alone, pending further validation. Dynamic imbalance induces RGC injury through dual mechanisms: mechanical stress on the lamina cribrosa (collagen fiber rupture, astrocyte activation) and metabolic dysfunction (ocular glymphatic clearance impairment, toxic waste accumulation), which ultimately converge on the activation of the programmed axonal degeneration (PAD) pathway—a conserved final common effector of RGC axon loss. Phase mismatch is the core pathological pattern in NTG. In contrast, high-tension primary open-angle glaucoma (POAG) is characterized mainly by amplitude mismatch and abnormal frequency, with potential coexistence and mutual influence of these mechanisms. Verifiable clinical (24-h IOP-CSFP synchronous monitoring) and animal experiments are proposed. This hypothesis may help explain unresolved clinical phenomena, provides novel diagnostic markers (a transient peak in TLCPD) and therapeutic strategies (CSFP regulation, glymphatic function enhancement, modulation of the PAD pathway), and opens new avenues for personalized glaucoma management.

## 1 Introduction

As the leading cause of irreversible blindness worldwide, glaucoma is characterized by the progressive loss of retinal ganglion cells (RGCs) and their axons as the core pathological change, ultimately resulting in irreversible visual field damage (1–3). Primary open-angle glaucoma (POAG) is the most common subtype, with its pathogenesis not yet fully elucidated (4). Elevated intraocular pressure (IOP) is widely recognized as the core risk factor, and reducing IOP remains the main clinical approach to slow disease progression (5). However, approximately 30–40% of POAG patients present with normal-tension glaucoma (NTG), meaning their IOP is consistently maintained within the normal range of 10–21 mmHg, yet they still develop typical glaucomatous optic neuropathy (GON) and visual field defects (6). This suggests that, in addition to static IOP, there are other key pathogenic factors involved.

The trans-lamina cribrosa pressure difference (TLCPD) is closely correlated with the pressure gradient between IOP and intracranial pressure (ICP), serving as a key mechanical indicator that bridges the intraocular and intracranial environments (7). As a sieve-like collagenous structure located at the posterior pole of the ocular wall, the lamina cribrosa is an indispensable pathway for RGC axons to pass from the high-pressure intraocular environment to the low-pressure retrobulbar cerebrospinal fluid (CSF) space. Its structural integrity and functional stability directly depend on the dynamic balance of TLCPD (8). Previous studies have confirmed that ICP is significantly lower in glaucoma patients, especially those with NTG, compared with healthy individuals, suggesting that low ICP may induce optic nerve damage by increasing TLCPD (9–11). However, the traditional “static TLCPD elevation” theory fails to explain the following clinical phenomena: some glaucoma patients exhibit disease progression despite having a normal static TLCPD; in some high-tension glaucoma patients, TLCPD returns to normal after IOP-lowering treatment, yet optic nerve damage persists (12).

ICP is a key determinant of cerebrospinal fluid pressure (CSFP) within the optic nerve sheath. While systemic studies often focus on ICP because it is clinically measurable, the biomechanical force that directly counteracts IOP at the lamina cribrosa is CSFP. Therefore, in the context of TLCPD homeostasis, CSFP is the critical intracranial counterpart to IOP. Emerging evidence suggests that not only absolute levels but also the dynamic fluctuations of CSFP (coupled with ICP dynamics) play a pivotal role in the pathogenesis of NTG (13).

In recent years, the discovery of the ocular glymphatic system has provided a new direction for investigating the pathogenesis of glaucoma. The ocular glymphatic system enables bidirectional exchange between CSF and intraocular fluid through the perivascular spaces surrounding the optic nerve, participating in the clearance of metabolic wastes (such as amyloid-*β*) (14, 15). Its function is driven by the pressure gradient between IOP and CSFP. Studies have shown that ocular glymphatic system dysfunction exists in glaucomatous animal models, characterized by impaired CSF entry into the perivascular spaces of the optic nerve, leading to the accumulation of metabolic wastes (16). Meanwhile, clinical studies have revealed that both IOP and CSFP exhibit circadian fluctuations, and such fluctuations are more pronounced in glaucoma patients (17).

**Figure 1:**
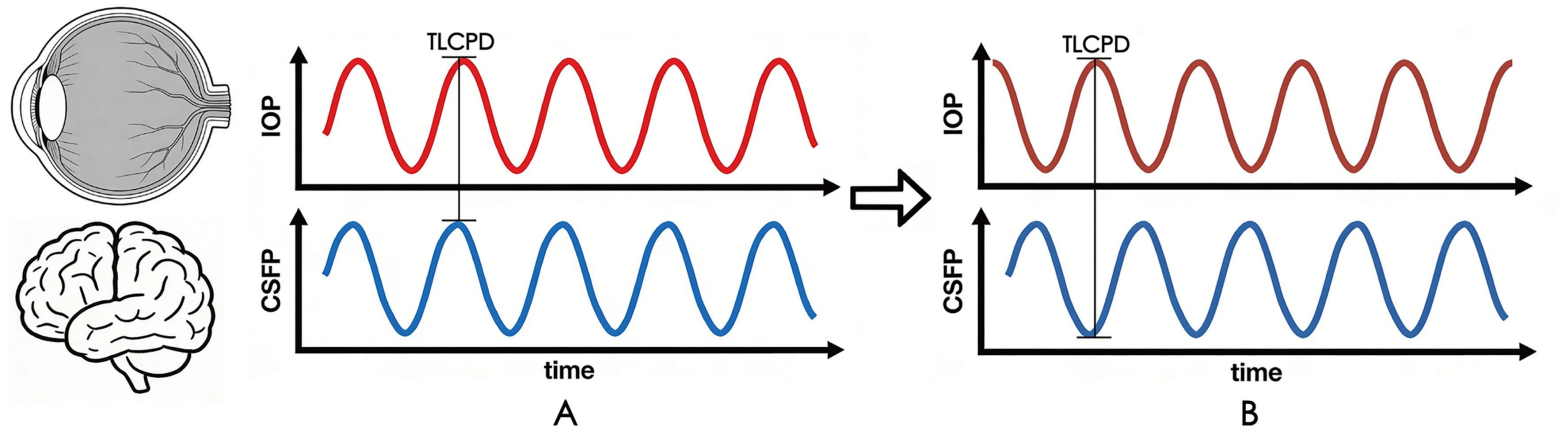
Schematic diagram illustrating the core role of pulsed synchrony regulation between intraocular pressure and cerebrospinal fluid pressure in the pathogenesis of glaucoma. Panel **(A)** shows that the pulsed fluctuations of IOP and CSFP are basically synchronized in healthy individuals, thereby creating a stable pressure difference. In contrast, panel **(B)** demonstrates that the pulsed fluctuations of IOP and CSFP are mismatched in patients with NTG, resulting in greater fluctuations in the pressure difference. Excessively high TLCPD may lead to compressive optic nerve damage, which is not easily detected by simple measurements of IOP and CSFP alone.

Based on the aforementioned research background, we propose the “dynamic imbalance of TLCPD” hypothesis: the optic nerve damage in glaucoma is not simply caused by the elevation of static TLCPD, but by the abnormal pulse synchrony between IOP and CSFP (i.e., the mismatched coordination of their fluctuation phases and amplitudes), which impairs the mechanical stability of the lamina cribrosa and the clearance function of the ocular glymphatic system, ultimately leading to the injury of RGC axons. In NTG, this dynamic imbalance is primarily manifested as the “mismatched superposition of IOP peaks and CSFP troughs.” Even if the static TLCPD is normal, the transient peak superposition can still induce irreversible optic nerve damage (Figure 1). This study will systematically elaborate on the theoretical basis and core mechanisms of this hypothesis, and propose verifiable experimental protocols, aiming to provide new insights into the interpretation of glaucoma pathogenesis and the optimization of therapeutic strategies, particularly for NTG.

## 2 Related research background and theoretical basis

### 2.1 Limitations of the static TLCPD theory

The traditional view holds that elevated TLCPD is the core mechanical mechanism underlying optic nerve damage in glaucoma (7). Under normal physiological conditions, the stable pressure difference between IOP and ICP in individuals collectively forms a static and stable TLCPD, providing an appropriate mechanical environment for RGC axons (18). When IOP increases or ICP decreases, TLCPD enlarges, which can cause posterior bulging of the lamina cribrosa and disorganization of collagen fibers. This in turn compresses RGC axons, impairs axoplasmic transport, and ultimately leads to RGC apoptosis (19).

Clinical studies support the role of low ICP in the pathogenesis of glaucoma: Berdahl et al. (9) found through lumbar puncture that ICP was significantly lower in POAG patients than in healthy controls, and ICP was also lower in NTG patients compared with healthy controls; a prospective study by Ren et al. (10) further confirmed that low ICP is an independent risk factor for POAG. In animal experiments, after reducing ICP in monkeys via implantation of a lumbar-peritoneal cerebrospinal fluid shunt, half of the animals developed typical glaucoma-like pathological changes, including thinning of the nerve fiber layer and enlargement of the optic cup (20), directly verifying the pathogenic effect of low ICP.

However, the static TLCPD theory has obvious limitations: Firstly, in Caucasian NTG patients, the average TLCPD calculated based on the initial cerebrospinal fluid opening pressure is only 3.2 ± 4.2 mmHg, and some patients even have relatively low TLCPD values (e.g., the lower range of −6.1 ~ 11.2 mmHg). Nevertheless, these patients all present with definite glaucomatous visual field defects and progressive disease (21); Secondly, after medical or surgical treatment, IOP in some high-tension glaucoma patients decreases to the normal range and static TLCPD returns to normal, but visual field defects continue to worsen (22); Finally, static TLCPD cannot explain the regional differences in glaucomatous damage. For example, some patients only exhibit inferior quadrant retinal nerve fiber layer loss, whereas static pressure differences should induce diffuse damage (23). These phenomena suggest that, in addition to static TLCPD, the dynamic fluctuation characteristics of IOP and ICP may be key pathogenic factors.

### 2.2 Circadian rhythm fluctuation characteristics of IOP and CSFP

Under physiological conditions, both IOP and CSFP exhibit significant circadian fluctuations, which are regulated by multiple factors including sleep–wake cycles, postural changes, and autonomic nervous system function (24–27). In healthy individuals, the peak mostly occurs at 04:00 a.m. and the trough at 08:00 p.m. (28). Meanwhile, studies have observed that CSFP also presents circadian fluctuations, with its peak likewise appearing at night (25). This indicates a certain degree of synchrony in the fluctuation phases of IOP and CSFP- i.e., CSFP increases slightly in parallel with elevated IOP, thereby maintaining a relatively stable TLCPD. In contrast, the IOP peak in POAG patients mostly emerges between 06:00 a.m. and 10:00 a.m., while the trough occurs between 10:00 p.m. and 02:00 a.m. This shift in peak timing suggests a potential mismatch in the timing of pressure pulses (25).

The fluctuation characteristics of IOP and CSFP undergo remarkable alterations in glaucoma patients: the amplitude of circadian IOP fluctuation may increase in POAG patients, with a possible prolongation of the peak duration (5); in NTG patients, although the IOP fluctuation amplitude remains within the normal range, the CSFP fluctuation amplitude decreases significantly, and a phase mismatch arises between the CSFP peak and the IOP peak. A study by Hou et al. (17) demonstrated that when ICP drops below the critical threshold, the synchronous fluctuation relationship between CSFP and IOP is disrupted, leading to stagnation of CSF flow in the subarachnoid space (SAS) surrounding the optic nerve and the formation of “cerebrospinal fluid compartment syndrome”, which may represent a key pathological change in NTG patients.

### 2.3 Pressure-dependent functional characteristics of the ocular glymphatic system

As an important pathway of the central nervous system, the ocular glymphatic system may extend its function to the optic nerve—via optic nerve-related fluid exchange pathways (such as the optic nerve subarachnoid space and interstitial space), it participates in the exchange between CSF and optic nerve interstitial fluid, and assists in the clearance of RGC-associated metabolic wastes (e.g., amyloid-*β*) (14). The function of this system relies on CSF flow driven by intracerebral arterial pulsation: CSF flows into the brain parenchyma along the perivascular spaces of intracerebral penetrating arteries, mixes with brain interstitial fluid, then carries metabolic wastes to flow back to the SAS along the perivascular spaces adjacent to intracerebral veins, and is ultimately drained into the bloodstream through arachnoid granulations or eliminated via cervical lymphatic vessels (15).

Studies have confirmed that the clearance function of the ocular glymphatic system is highly sensitive to the dynamic changes of pressure gradients. Mathieu et al. (16) found in the DBA/2 J glaucoma mouse model that the process of CSF entering the perivascular spaces of the optic nerve was blocked, leading to the accumulation of amyloid-*β* in the lamina cribrosa region. This obstruction is directly associated with the abnormal pressure gradient caused by the failure of CSFP to increase synchronously following IOP elevation. This study suggests that the synchronous fluctuation of IOP and CSFP is crucial for maintaining the function of the ocular glymphatic system, whereas the loss of such synchrony may result in metabolic waste accumulation and subsequent RGC damage.

## 3 Core mechanisms of the “dynamic TLCPD imbalance” hypothesis

### 3.1 Core connotation of the hypothesis

The core proposition of the “dynamic TLCPD imbalance” hypothesis is that optic nerve damage in glaucoma may not be solely induced by the elevation of the absolute value of static TLCPD but may instead arise from the abnormal pulse synchrony between IOP and CSFP. This hypothesis emphasizes that the temporal coordination and relative fluctuation characteristics of the two pressures, rather than their individual static values or isolated fluctuations, determine the integrity of the optic nerve head (ONH). Specifically, dynamic imbalance manifests in three interrelated forms, with distinct pathological implications for different glaucoma subtypes:

Fluctuation phase mismatch: Temporal overlap between the IOP peak and the CSFP trough leads to a transient but biologically impactful elevation of the TLCPD peak (defined as “transient peak TLCPD”), which exceeds the adaptive capacity of the lamina cribrosa (LC) and ocular glymphatic system. This form is the pathological hallmark of normal-tension glaucoma (NTG), where static TLCPD remains within the physiological range, but dynamic asynchrony induces progressive damage.
Fluctuation amplitude mismatch: The amplitude of IOP fluctuation is disproportionately larger than that of CSFP fluctuation, resulting in a TLCPD fluctuation range that surpasses the physiological tolerance limit of the ONH. This form is predominant in high-tension primary open-angle glaucoma (POAG), often synergizing with elevated static TLCPD to accelerate damage.
Abnormal fluctuation frequency: Pathological high-frequency oscillations of IOP or CSFP disrupt the mechanical homeostasis of LC collagen fibers and the continuity of axoplasmic transport, preventing the ONH from adapting to pressure changes. This form may coexist with an amplitude mismatch in severe or progressive POAG.

**Figure 2:**
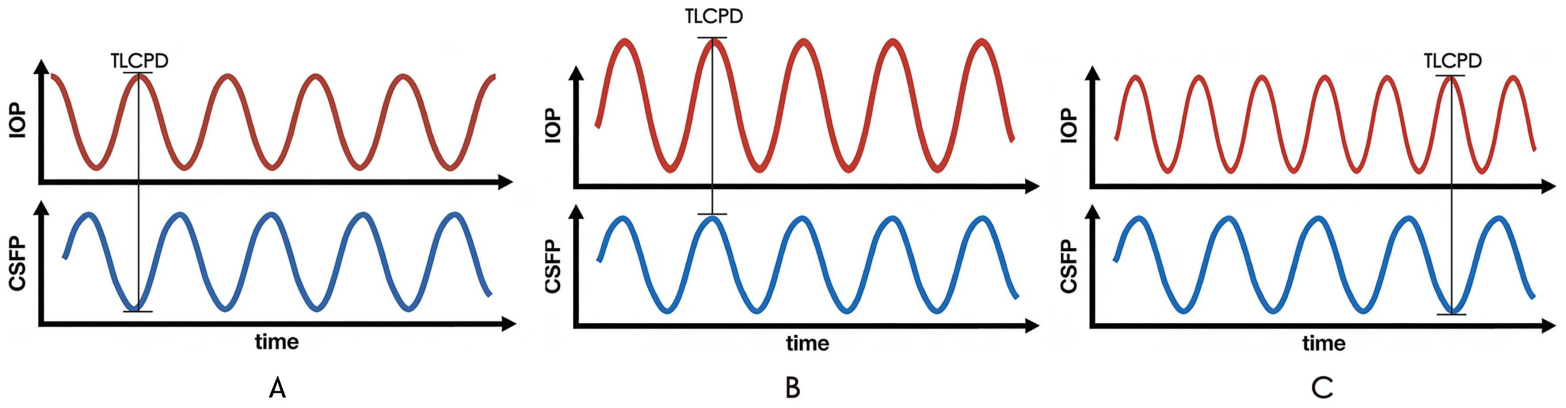
Schematic diagram of three core patterns of dynamic translaminar cribriform pressure difference (TLCPD) imbalance. Panel **(A)** corresponds to Figure 1B and represents “fluctuation phase mismatch.” Panel **(B)** illustrates “fluctuation amplitude mismatch,” while panel **(C)** depicts “abnormal fluctuation frequency.” Each pattern reflects distinct abnormal pulse synchrony between intraocular pressure (IOP) and cerebrospinal fluid pressure (CSFP), which underlies the pathogenesis of different glaucoma subtypes.

To intuitively distinguish the three core patterns of dynamic TLCPD imbalance and their characteristics, a schematic diagram is provided in Figure 2.

#### 3.1.1 Core innovations of the hypothesis vs. classical IOP fluctuation theory

The dynamic TLCPD imbalance hypothesis represents a fundamental departure from classical IOP fluctuation theory, which attributes glaucoma damage solely to excessive IOP variability (5, 29). The core innovations are threefold:

Paradigm shift from univariate to bivariate regulation: Classical theory frames IOP as the sole pressure determinant of ONH health, treating CSFP as a static, negligible factor. In contrast, our hypothesis identifies TLCPD (the interaction of IOP and CSFP) as the core regulatory variable, emphasizing that the synchrony of their fluctuations is as critical as their absolute values. This explains why many patients with high IOP variability do not develop glaucoma (due to synchronous CSFP buffering) and why NTG patients with “normal” IOP variability still progress (due to CSFP asynchrony).
Recognition of CSFP as a protective factor: Classical theory does not account for CSFP’s physiological role in buffering IOP fluctuations. Our hypothesis highlights that CSFP’s circadian synchrony with IOP is a key protective mechanism—one that is disrupted in NTG. This shifts the therapeutic focus from reducing IOP variability alone to restoring IOP-CSFP synchrony, addressing an unmet need in NTG management.
Explanation of the “normal-tension” phenotype: Classical IOP fluctuation theory cannot reconcile the paradox of NTG (progressive damage with normal static IOP). Our hypothesis resolves this by demonstrating that NTG damage is driven by the loss of CSFP buffering, rather than by extreme IOP elevation. This provides a unifying mechanistic framework for understanding why IOP-lowering therapy alone is often ineffective in NTG—such treatments may reduce IOP peaks but fail to correct CSFP-IOP asynchrony, leaving the transient peak TLCPD intact.

#### 3.1.2 Clinical heterogeneity and overlap of dynamic imbalance patterns

While the “phase mismatch” and “amplitude/frequency mismatch” are proposed as the pathological hallmarks of NTG and high-tension POAG (HTG), respectively, it is critical to acknowledge that these patterns are not mutually exclusive in clinical practice. The dynamic TLCPD imbalance hypothesis should be viewed as a spectrum rather than a rigid binary classification.

Clinical observations suggest significant heterogeneity: (1) NTG patients may exhibit increased IOP fluctuation amplitudes secondary to progressive optic nerve damage or systemic factors, meaning they can present with a combination of phase mismatch and amplitude mismatch; (2) HTG patients, especially those with poorly controlled circadian rhythms, may also suffer from phase misalignment between IOP and CSFP, which can accelerate disease progression independent of elevated static IOP.

The proposed classification refers to the dominant or initiating pathological factor in each subtype: NTG is primarily driven by the loss of CSFP buffering (phase mismatch) even with normal IOP amplitude, while HTG is primarily driven by excessive IOP variability (amplitude/frequency mismatch) that overwhelms CSFP compensation. This nuanced view accommodates clinical complexity and explains why some patients respond poorly to conventional IOP-lowering therapy—their underlying dynamic imbalance pattern may be mixed or dominated by an unaddressed mechanism (e.g., CSFP dysregulation in HTG patients with phase mismatch).

### 3.2 Dual mechanisms of optic nerve damage induced by dynamic imbalance

#### 3.2.1 Mechanical damage mechanism: dynamic mechanical stress on the lamina cribrosa

As an elastic structure composed of collagen fibers and astrocytes, the mechanical stability of the lamina cribrosa depends on the dynamic balance of TLCPD (8). Under physiological conditions, the synchronous fluctuations of IOP and CSFP render the mechanical stress borne by the lamina cribrosa in a periodically reversible state, without inducing structural damage. In the event of dynamic imbalance, the transient peak TLCPD can generate tensile and shear stresses exceeding the elastic limit of the local lamina cribrosa, leading to collagen fiber rupture and astrocyte activation (30).

Upon activation, astrocytes release matrix metalloproteinases (MMPs), which further degrade the collagen fibers of the lamina cribrosa and result in permanent structural relaxation of the lamina cribrosa (31). Meanwhile, the transient high-pressure difference can directly compress the axons of RGCs, causing stagnation of axoplasmic transport, which is characterized by impeded delivery of neurotrophic factors (e.g., brain-derived neurotrophic factor), and ultimately triggers RGC apoptosis (32). Studies have confirmed that mechanical damage to the lamina cribrosa has a cumulative effect; even if a single episode of transient peak TLCPD does not induce obvious structural changes, long-term recurrent dynamic imbalance can still lead to progressive thinning of the lamina cribrosa and continuous loss of RGCs (33).

#### 3.2.2 Metabolic damage mechanism: dysfunction of the ocular glymphatic system clearance

As mentioned earlier, the function of the ocular glymphatic system relies on CSF flow driven by intracerebral arterial pulsation (15). When phase mismatch occurs between IOP and CSFP fluctuations, the pressure gradient in the perivascular spaces undergoes transient reversal, which results in stagnation of bidirectional exchange between CSF and intraocular fluid (16). This stagnant state leads to the accumulation of toxic metabolic products from RGCs (such as amyloid-*β* and tau protein) in the lamina cribrosa region (34).

The accumulation of amyloid-*β* can damage RGCs through multiple pathways: direct disruption of cell membrane integrity, induction of oxidative stress responses, and activation of microglia-mediated inflammatory reactions, etc. (35). Studies have shown that the deposition of amyloid-β in the optic nerve tissues of NTG patients is significantly higher than that in healthy individuals, and the deposition sites are highly consistent with the areas of RGC loss (36). Meanwhile, the reversal of the pressure gradient can cause stenosis of the perivascular spaces, which further aggravates the obstruction of CSF flow, forming a vicious cycle of “accumulation-damage-stenosis,” and ultimately accelerates RGC death and optic nerve fibrosis (37).

**Figure 3:**
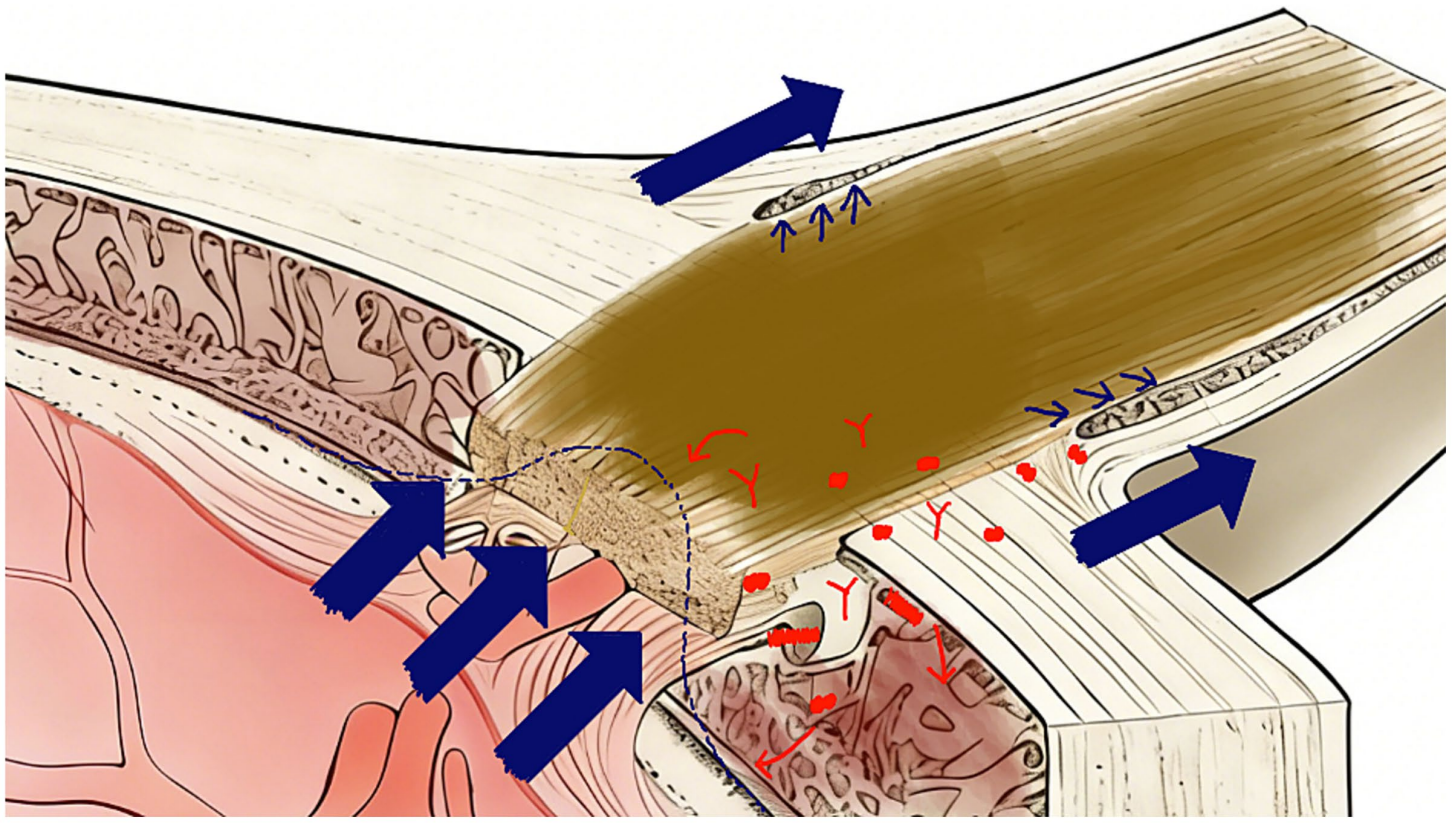
Schematic diagram illustrating the synergistic vicious cycle of mechanical and metabolic damage in glaucomatous optic neuropathy. Dynamic translaminar cribriform pressure difference (TLCPD) imbalance initiates mechanical stress, which impairs ocular glymphatic function (metabolic damage), as indicated by the blue arrows. Accumulation of metabolic waste (red sign) further compromises the mechanical tolerance of the optic nerve head, thereby forming a vicious circle that accelerates retinal ganglion cell loss.

In summary, the mechanical and metabolic damage mechanisms are not mutually exclusive parallel processes but function as a temporally ordered, synergistic unit that forms a self-reinforcing vicious cycle. Mechanistically, mechanical stress acts as the initial trigger: acute dynamic TLCPD imbalance (e.g., peak-trough superposition) first disrupts the lamina cribrosa architecture and compromises the patency of perivascular spaces. This structural compromise then impairs ocular glymphatic clearance, leading to the accumulation of metabolic waste and neuroinflammatory mediators—the secondary amplifying step. Critically, this metabolic dysfunction further reduces the mechanical tolerance of optic nerve head astrocytes and collagen fibers, making the tissue hypersensitive to subsequent episodes of dynamic imbalance. This vicious cycle explains the chronic and progressive nature of glaucomatous damage observed clinically, even in NTG patients with seemingly “normal” static pressure. To clarify this complex causal relationship, a schematic illustration of the synergistic vicious cycle is provided in Figure 3.

#### 3.2.3 PAD pathway: the final common effector of dynamic imbalance

The mechanical and metabolic vicious cycle described above ultimately converges on the activation of the programmed axonal degeneration (PAD) pathway, representing the final common effector responsible for progressive RGC axon loss in both HTG and NTG. In HTG, the excessive mechanical stress from amplitude/frequency mismatch initiates a distal-to-proximal axonopathy that phenocopies slow Wallerian degeneration. This process is not a passive decay but an actively regulated program, as evidenced by the significant neuroprotection afforded by the slow Wallerian degeneration allele in glaucoma models (38). In NTG, where mechanical stress is subtler but metabolic impairment (e.g., glymphatic clearance failure, mitochondrial dysfunction) is predominant, the PAD pathway is triggered through IOP-independent mechanisms. Central to this pathway is the dysregulation of NAD^+^ metabolism. Metabolic waste accumulation and mitochondrial dysfunction, as consequences of dynamic TLCPD imbalance, elevate the NMN/NAD^+^ ratio (39). This imbalance activates Sterile Alpha and TIR Motif Containing 1 (SARM1), a key pro-degenerative protein in the PAD cascade. Even partial reduction of SARM1 activity is sufficient to delay axonal degeneration *in vivo* (40). Collectively, our dynamic imbalance hypothesis provides a potential unifying upstream framework that may explain how distinct glaucomatous insults. Converge on the conserved PAD pathway to drive irreversible neurodegeneration.

### 3.3 Differences in dynamic imbalance between NTG and high-tension POAG

There may be significant differences in the characteristics of dynamic imbalance between NTG and high-tension POAG, which may in turn contribute to the differences in their clinical phenotypes:

NTG: The static TLCPD remains normal, but the mismatched superposition of IOP peaks and CSFP troughs results in elevated transient peak TLCPD with a relatively low fluctuation frequency. Since the transient peaks mainly act on the vulnerable regions of the lamina cribrosa (e.g., the inferior quadrant), NTG patients typically present with localized retinal nerve fiber layer loss and visual field defects (such as arcuate scotomas) (41).
High-tension POAG: The static TLCPD is elevated, accompanied by an increased amplitude and frequency of IOP fluctuations, while the amplitude of CSFP fluctuations remains relatively normal, leading to a marked expansion of the TLCPD fluctuation range. Sustained high-frequency and large-amplitude fluctuations induce diffuse damage to the lamina cribrosa, and thus, patients mostly exhibit diffuse retinal nerve fiber layer thinning and extensive visual field defects (42).

This difference also explains why NTG patients respond poorly to simple IOP-lowering treatment: although IOP-lowering therapy can reduce IOP peaks, it fails to correct the mismatch between CSFP troughs and IOP peaks. As a result, the transient peak TLCPD persists, leading to disease progression (29).

## 4 Verifiability of the hypothesis

### 4.1 Clinical verification protocol

#### 4.1.1 24-h simultaneous monitoring of IOP and CSFP

To explore the dynamic relationship between IOP and CSFP over a 24-h cycle, we propose an experimental approach that incorporates continuous, simultaneous monitoring of both parameters. This approach aims to understand better the potential temporal misalignment or amplitude discrepancies between IOP and CSFP that may contribute to transient peaks in TLCPD. By monitoring IOP and CSFP in real time, we can capture fluctuations in both parameters that may not be evident in static measurements, providing valuable insights into their dynamic interactions (43).

However, it is important to acknowledge the current limitations in accurately measuring CSFP and IOP over extended periods. While invasive techniques, such as lumbar puncture for CSFP measurement, are the gold standard, their clinical applicability is limited. Non-invasive methods, such as transcranial Doppler or ocular sonography, offer potential alternatives but have yet to be fully validated for detecting subtle circadian fluctuations in CSFP or for ensuring accurate synchronization between IOP and CSFP. Given these technological constraints, this experimental design should be exploratory, aiming to generate hypotheses about the dynamic interaction between IOP and CSFP rather than as a definitive solution for monitoring these pressures in clinical practice.

In future studies, improved sensor technologies and multimodal approaches, such as combining transcranial Doppler with novel non-invasive CSFP estimation techniques, will be essential to provide a more comprehensive picture of the pressure dynamics involved. Such advancements will be crucial for validating the “dynamic TLCPD imbalance” hypothesis and further elucidating the role of IOP-CSFP synchronization in glaucoma pathogenesis.

A total of 30 NTG patients, 30 POAG patients, and 30 healthy controls were enrolled, and the following indicators were calculated:

Fluctuation phase difference: the time difference between the IOP peak and the CSFP peak.
Transient peak TLCPD: the difference between the IOP peak and the corresponding CSFP value at the same time point;
Fluctuation amplitude ratio: the ratio of the IOP fluctuation amplitude to the CSFP fluctuation amplitude.

#### 4.1.2 Detection of optic nerve axoplasmic flow and metabolic wastes

Optical coherence tomography angiography (OCTA) was used to measure the width of the perivascular spaces of the optic nerve for evaluating the function of the ocular glymphatic system; lumbar puncture was performed to detect the levels of amyloid-*β* and tau protein in the cerebrospinal fluid. The subjects involved in the above monitoring were included to analyze the correlations between the transient peak TLCPD and the following indicators: the width of perivascular spaces, the cerebrospinal fluid amyloid-β 42/40 ratio, and the annual reduction rate of retinal nerve fiber layer thickness.

### 4.2 Animal experiment verification protocol

Given the current research gap in validating IOP-CSFP synchrony modulation, the following protocol is proposed as a preliminary exploratory framework rather than a finalized procedure. Significant optimization through preliminary experiments is required to address feasibility, variability, and reproducibility challenges.

#### 4.2.1 Establishment of animal models with dynamic imbalance

To avoid confounding effects from pre-existing glaucomatous pathology, C57BL/6 mice (8–10 weeks old) were selected as the sole experimental subjects across all groups. The modeling strategies below are derived from rational inference based on mature techniques in circadian rhythm regulation and glaucoma research. Still, their effectiveness in inducing target dynamic imbalance remains to be verified.

##### 4.2.1.1 Experimental group 1 (phase mismatch model)

Serotonin was selected as the intervention agent based on direct evidence of modulation of CSF secretion. Serotonin was dissolved in sterile artificial cerebrospinal fluid (aCSF) to a final concentration of 100 μM. Mice underwent stereotaxic surgery 1 week before intervention to implant a guide cannula (C315GS-5, Plastics One) targeting the lateral ventricle (coordinates: AP: −0.5 mm, ML: ±1.0 mm, DV: −2.0 mm relative to bregma) for repeated intracerebroventricular (ICV) injections. The intervention involved ICV administration of 5 μL serotonin solution every 48 h at 16:00 (4 h before dark cycle onset) for 12 weeks.

Rationale: A published study confirmed that central serotonin administration (100 μM) reduces CSF secretion rate by ~25% in rats (25), which is expected to lower CSFP during the subsequent light cycle (06:00–10:00)—coinciding with the natural IOP peak of C57BL/6 mice. This temporal overlap aims to simulate the core NTG phenotype: normal static TLCPD but mismatched IOP-CSFP fluctuation phases.

Key uncertainties: Whether the 100 μM concentration and 48-h injection interval can stably induce CSFP reduction in mice; long-term safety of repeated ICV injections (e.g., cannula blockage, neuroinflammation); and whether the resulting phase mismatch can induce NTG-like optic nerve damage. Preliminary experiments will optimize injection frequency (48/72 h) and verify CSFP dynamics via optic nerve sheath diameter monitoring.

##### 4.2.1.2 Experimental group 2 (amplitude mismatch model)

Intermittent IOP elevation was attempted via anterior chamber injection of balanced salt solution (BSS). Under anesthesia, a 26-gauge syringe with a beveled tip was used to inject an initial volume of 5 μL BSS into the anterior chamber. Injections were administered every 48 h at random times during the day (09:00–17:00) to avoid circadian entrainment.

Rationale: Although no prior studies have used the method of injecting 5 μL BSS into the anterior chamber to establish a high IOP animal model, this approach is specifically designed to match the Experimental group 1 (phase mismatch model). A key advantage of this model lies in the artificial controllability of both the magnitude and frequency of IOP elevation. Furthermore, it enables the frequency of invasive procedures to be consistent with that of group 1, ensuring methodological uniformity across the experimental design.

Key Uncertainties: Feasibility of maintaining stable IOP fluctuation amplitude without inducing permanent ocular damage (e.g., corneal endothelial dysfunction, iritis); inter-individual variability in IOP response to injection. Preliminary experiments will optimize injection volume (3, 5, or 8 μL) and frequency, with histopathological assessment of ocular tissues.

#### 4.2.2 Model validation indicators

Mechanical indicators: Atomic force microscopy was used to detect the elastic modulus of the lamina cribrosa collagen fibers for evaluating the degree of mechanical damage.

Functional indicators: Fluoro-Gold retrograde labeling was used to detect the survival rate of RGCs for evaluating axoplasmic transport function.

Metabolic indicators: Immunofluorescence staining was performed to detect the deposition of amyloid-*β* in the optic nerve tissues.

IOP monitoring: IOP was measured at fixed time points using a TonoLab rebound tonometer (iCare, Vantaa, Finland) in both groups.

### 4.3 Intervention experiment verification

Twenty NTG patients with relatively high transient peak TLCPD in clinical monitoring were selected and treated with CSFP synchronous regulation intervention: intranasal administration (e.g., vasopressin receptor agonists) was used to regulate the circadian rhythm of CSFP, so that the CSFP peak was synchronized with the IOP peak. The intervention lasted for 6 months, and the following indicators were monitored during the period: changes in transient peak TLCPD; changes in retinal nerve fiber layer thickness; progression of visual field defects.

## 5 Discussion

The “dynamic TLCPD imbalance” hypothesis proposed in this study provides a novel interpretation of the pathogenesis of glaucoma, based on the circadian rhythm characteristics of IOP and CSFP, the pressure-dependent function of the glymphatic system, and the differences in clinical phenotypes of glaucoma reported in previous studies. The core innovation of this hypothesis lies in breaking through the limitations of the traditional “static TLCPD elevation” theory and emphasizing that the abnormal pulse synchrony between IOP and CSFP is the key factor leading to optic nerve damage, which, in particular, offers a reasonable explanation for the pathogenesis of NTG.

### 5.1 Consistency between the hypothesis and existing research

This hypothesis is highly consistent with the findings of multiple clinical and basic studies, supporting its biological plausibility: First, the amplitude of circadian ICP fluctuation is significantly reduced in NTG patients, resulting in the failure of CSFP to increase synchronously with IOP and thus the formation of phase mismatch (17), which is consistent with the characteristics of dynamic imbalance in NTG proposed by the hypothesis; Second, the time of occurrence of ocular glymphatic system dysfunction in glaucomatous animal models coincides with the onset of abnormal IOP-CSFP synchrony (16), suggesting that dynamic imbalance is an upstream event triggering metabolic damage; Third, this hypothesis may help explain the clinical phenomena that cannot be fully elucidated by the traditional theory: why some NTG patients with normal static TLCPD still experience disease progression (due to elevated transient peak TLCPD); why some high-tension POAG patients may still progress after receiving IOP-lowering treatment (because the fluctuation amplitude and frequency remain uncorrected); and why glaucomatous damage often presents with regionality (due to selective damage to the vulnerable regions of the lamina cribrosa).

### 5.2 Clinical significance of the hypothesis

#### 5.2.1 Optimizing the diagnosis of glaucoma

At present, the diagnosis of glaucoma mainly relies on static IOP measurement and structural/functional assessment of the optic nerve, but these indicators have low sensitivity for the early diagnosis of NTG (44). Based on this hypothesis, 24-h simultaneous IOP-CSFP monitoring has the potential to serve as a novel indicator for the early diagnosis of NTG, pending clinical validation. In particular, the transient peak TLCPD and fluctuation phase difference may act as more sensitive diagnostic markers than static IOP. In addition, combining the width of perivascular spaces detected by OCTA and the level of metabolic wastes in cerebrospinal fluid can further improve diagnostic accuracy, enabling early intervention for glaucoma.

#### 5.2.2 Expanding therapeutic strategies

The current core of glaucoma treatment is to reduce static IOP, but NTG patients respond poorly to simple IOP-lowering therapy (29). Based on the “dynamic TLCPD imbalance” hypothesis, future treatments can be expanded in the following aspects:

Synchronous regulation of CSFP: Regulate the circadian rhythm of CSFP through drugs (e.g., vasopressin receptor agonists) or implantable cerebrospinal fluid pumps to achieve synchronous fluctuation between CSFP and IOP (45);
Improving ocular glymphatic system function: Dilate the perivascular spaces through drugs (e.g., AQP4 agonists) to enhance the clearance of metabolic wastes (46);
Protecting the mechanical stability of the lamina cribrosa: Inhibit the degradation of lamina cribrosa collagen fibers through drugs (e.g., MMP inhibitors) to improve its mechanical tolerance (47).

These therapeutic strategies target the proposed core links of dynamic imbalance, which may provide new therapeutic directions for refractory glaucoma, such as NTG, upon further experimental and clinical verification.

### 5.3 Limitations of the hypothesis and future research directions

This hypothesis still has certain limitations, and various studies to verify this hypothesis face certain difficulties: First, as a hypothesis-driven work, retrieving direct supporting literature is inherently challenging, and there is currently a lack of direct evidence confirming that the phase mismatch between IOP and CSFP can lead to elevated transient peak TLCPD, which needs to be further verified through 24-h synchronous monitoring; Second, the molecular mechanism by which dynamic imbalance induces ocular glymphatic system dysfunction has not been fully clarified, and relevant preclinical studies supporting intermediate links are relatively scarce; Third, the hypothesis does not consider the regulatory role of genetic factors in dynamic imbalance, such as whether genetic variations are affecting the synchronous regulation of IOP and CSFP.

Future research should focus on the following directions: conducting large-sample, multi-center clinical studies to verify the correlation between transient peak TLCPD and glaucoma progression; using single-cell sequencing technology to decipher the response mechanism of lamina cribrosa cells to dynamic mechanical stress; and developing non-invasive and accurate CSFP monitoring technologies to provide tools for clinical intervention. In addition, targeted drugs and intervention devices developed based on this hypothesis need to be verified for their efficacy and safety through randomized controlled clinical trials.

## 6 Conclusion

In summary, this study proposes the “dynamic TLCPD imbalance” hypothesis, which provides an innovative potential reinterpretation of glaucoma pathogenesis by emphasizing abnormal pulse synchrony between IOP and CSFP as a potential core pathogenic factor, breaking the limitations of the traditional “static TLCPD elevation” theory. The hypothesis posits that dynamic imbalance manifests in three forms—phase mismatch, amplitude mismatch, and abnormal frequency—with distinct characteristics in NTG and high-tension POAG and induces optic nerve damage through dual mechanisms of mechanical stress on the lamina cribrosa and metabolic dysfunction of the ocular glymphatic system.

Supported by existing clinical and basic research, this hypothesis reasonably explains several unresolved clinical phenomena, such as disease progression in NTG patients with normal static TLCPD and poor response to only IOP-lowering therapy. Moreover, it provides novel directions for clinical practice: 24-h synchronous IOP-CSFP monitoring and combined detection of perivascular space width and metabolic markers offer potential for optimizing glaucoma diagnosis, especially for early NTG. Targeted interventions, including CSFP rhythm regulation, ocular glymphatic function enhancement, and lamina cribrosa protection, may address the unmet therapeutic needs of refractory glaucoma.

While the hypothesis requires validation through large-sample clinical studies, refined animal experiments, and non-invasive CSFP monitoring technology development, its emphasis on the dynamic interaction between intraocular and intracranial pressure systems opens new avenues for understanding glaucoma pathogenesis. Ultimately, this work aims to lay the foundation for a potential transition from static IOP-centered diagnosis and treatment to dynamic balance-targeted strategies, which may facilitate individualized management and improve outcomes for glaucoma patients worldwide if validated.
